# Atypical Carcinoid Syndrome in a Patient Presenting With Pericarditis and Supraventricular Tachycardia: A Case Report

**DOI:** 10.7759/cureus.30052

**Published:** 2022-10-08

**Authors:** Zahid Khan, George Besis, Niket Patel

**Affiliations:** 1 Acute Medicine, Mid and South Essex NHS Foundation Trust, Southend-on-Sea, GBR; 2 Cardiology and General Medicine, Barking, Havering and Redbridge University Hospitals NHS Trust, London, GBR; 3 Cardiology, Royal Free Hospital, London, GBR

**Keywords:** chemotherapy agents, landmark-guided pericardiocentesis, acute pericardial effusion, transthoracic echocardiogram, anginal chest pain, normal coronary angiogram, paroxysmal supraventricular tachycardia, pulmonary carcinoid tumor, mediastinal carcinoid tumor, atypical carcinoid

## Abstract

Atypical carcinoids are more uncommon than typical carcinoids, and carcinoid syndrome in general is quite rare. Mediastinal atypical carcinoid is a rare neuroendocrine tumor (NET) that spreads aggressively and rapidly. Morphologically, neuroendocrine tumors are classified into typical carcinoid, atypical carcinoid, small cell carcinoma, and large cell neuroendocrine carcinoma, and the latter two are high-grade tumors. The incidence of atypical carcinoid is rare, and the prognosis is poor, which makes larger trials difficult. It may affect the liver, lungs, or mediastinum with or without metastasis. We present a case of a 47-year-old male patient who presented with chest pain and was found to be in supraventricular tachycardia (SVT) on initial presentation to the hospital. A repeat electrocardiogram (ECG) showed widespread ST-segment elevation. A bedside echocardiogram showed a moderate pericardial effusion, and the patient underwent a coronary angiogram, which showed normal coronary arteries. A computed tomography pulmonary angiogram (CTPA) showed a right mediastinal mass, and the patient was referred to oncology following a discussion in a multidisciplinary team (MDT) meeting. He was commenced on neoadjuvant chemotherapy and has been followed up since in the outpatient clinic. This case is unique due to the initial presentation of supraventricular tachycardia and pericardial effusion.

## Introduction

Carcinoid tumors are mostly found in the gastrointestinal tract (GIT) and are neuroendocrine tumors (NETs) derived from enterochromaffin or Kulchitsky cells [1]. They secrete serotonin or other chemicals into the bloodstream that gets distributed to the various parts of the body through the bloodstream. NETs arise in most organs of the body, including the lung, thymus, GIT, and ovary [2]. According to the WHO classification of NETs in 2015, lung NETS are categorized into four histologic variants: well-differentiated, low-grade typical carcinoid; well-differentiated, intermediate-grade atypical carcinoid; slightly differentiated, high-grade large cell neuroendocrine carcinoma; and slightly differentiated, high-grade small cell lung carcinoma [3,4]. The prognosis for atypical carcinoid is very poor, and larger study trials are not possible due to the rarity of the condition and the fact that patients diagnosed with this condition are in different parts of the world. Typical carcinoid most commonly occurs in the GIT, followed by the lungs, and it has been reported to develop synchronously in both lungs [4]. NETs account for only 5% of the total newly diagnosed lung malignancies [4].

Well-differentiated lung NETs (typical and atypical carcinoid) account for about 27% of all NETs and commonly develop in non-smokers or light smokers. They are capable of lower mitotic rates, necrosis, and genetic abnormalities in comparison to high-grade NETs [4]. NETs are sometimes also classified into secretory and non-secretory NETs. Atypical carcinoid has a higher male preponderance, and the male/female ratio is 3:1. The average age of presentation for atypical carcinoid is about 60 years, although most cases present between the age of 39 and 60 years [5]. In contrast to most NETs originating from the GIT and lungs, mediastinal NETs are very aggressive and metastasize rapidly. The exact origin of mediastinal NETs is still debatable and is not clear, although thymic tumors are the most found NETs, accounting for 2%-4% of all mediastinal tumors, and are mainly found in the anterosuperior mediastinum [6].

We present a case of an atypical mediastinal carcinoid patient who presented with supraventricular tachycardia (SVT), and electrocardiogram (ECG) features were suggestive of acute pericarditis. A coronary angiogram showed normal coronary arteries, and computed tomography (CT) of the chest showed a right mediastinal mass. Biopsy findings were consistent with the diagnosis of an atypical carcinoid tumor.

## Case presentation

A 47-year-old male patient presented with a three-day history of shortness of breath (SOB) and chest pain. Past medical history (PMH) included smoking and hypertension. Family history was significant for diabetes and hypertension only. His regular medications included amlodipine for hypertension. Vital signs on initial presentation were as follows: blood pressure (BP) of 135/98 mmHg, heart rate (HR) of 78 bpm, respiratory rate (RR) of 20 bpm, temperature of 37.2°C, and oxygen saturation (SpO_2_) of 97% on room air. His total body weight was 78 kg. Physical examination was normal, apart from mildly reduced air entry in the right lung, and the patient did not have any pericardial rub. The laboratory test results are shown in Table 1. Electrocardiogram (ECG) with paramedics showed supraventricular tachycardia, and ECG in the emergency department showed widespread ST-segment elevation.

**Table 1:** Laboratory test result trend of the patient over a five-week period

Test	Day 1	Day 3	Day 7	Day 10	Day 33	Reference value
White cell count	7.58 × 10^9^/L	8.51 × 10^9^/L	13.06 × 10^9^/L	16.77 × 10^9^/L	6.81 × 10^9^/L	3.5-11 × 10^9^/L
Neutrophil	5.67 × 10^9^/L	4.47 × 10^9^/L	9.16 × 10^9^/L	10.85 × 10^9^/L	3 × 10^9^/L	1.7-7.5 × 10^9^/L
Hemoglobin	114 g/L	115 g/L	128 g/L	122 g/L	124 g/L	135-170 g/L
Platelet	546 × 10^9^/L	564 × 10^9^/L	565 × 10^9^/L	637 × 10^9^/L	387 × 10^9^/L	140-400 × 10^9^/L
Urea	4.2 mmol/L	5.1 mmol/L	6.3 mmol/L	4.7 mmol/L	5 mmol/L	2.9-8.2 mmol/L
Creatinine	79 umol/L	86 umol/L	75 umol/L	72 umol/L	72 umol/L	66-112 umol/L
Sodium	133 mmol/L	133 mmol/L	132 mmol/L	129 mmol/L	137 mmol/L	135-145 mmol/L
Potassium	4.7 mmol/L	5.3 mmol/L	5.2 mmol/L	5.6 mmol/L	5.2 mmol/L	3.5-5.1 mmol/L
C-reactive protein	184 mg/L	135 mg/L	99 mg/L	266 mg/L	213 mg/L	0-5 mg/L
Troponin	9 ng/L	10 ng/L	8 ng/L	-	-	<14 ng/L
D-dimer	-	2,977 ng/mL	-	1,288 ng/mL	-	0-400 ng/mL
N-terminal pro-brain natriuretic peptide	512 ng/L	452 ng/L	331 ng/L	317 ng/L	65 ng/L	<400 ng/L
Bilirubin	22 umol/L	20 umol/L	21 umol/L	19 umol/L	6 umol/L	0-21 umol/L
Alanine aminotransferase	192 unit/L	151 unit/L	103 unit/L	57 unit/L	21 unit/L	10-50 unit/L
Aspartate aminotransferase	94 unit/L	58 unit/L	36 unit/L	34 unit/L	32 unit/L	10-50 unit/L
Alkaline phosphatase	180 unit/L	186 unit/L	163 unit/L	150 unit/L	145 unit/L	0-129 unit/L

A bedside echocardiogram showed a moderate pericardial effusion. The patient underwent an emergency coronary angiogram that showed mild disease in the left anterior descending artery and dominant right coronary artery but otherwise unobstructed coronary arteries (Videos 1-3).

**Video 1:** Coronary angiogram showing normal right coronary artery

**Video 2:** Coronary angiogram showing normal left-sided coronary arteries including the left main stem, left anterior descending artery, and left circumflex artery

**Video 3:** Coronary angiogram of the right coronary artery showing unobstructed coronary artery

**Figure 1:**
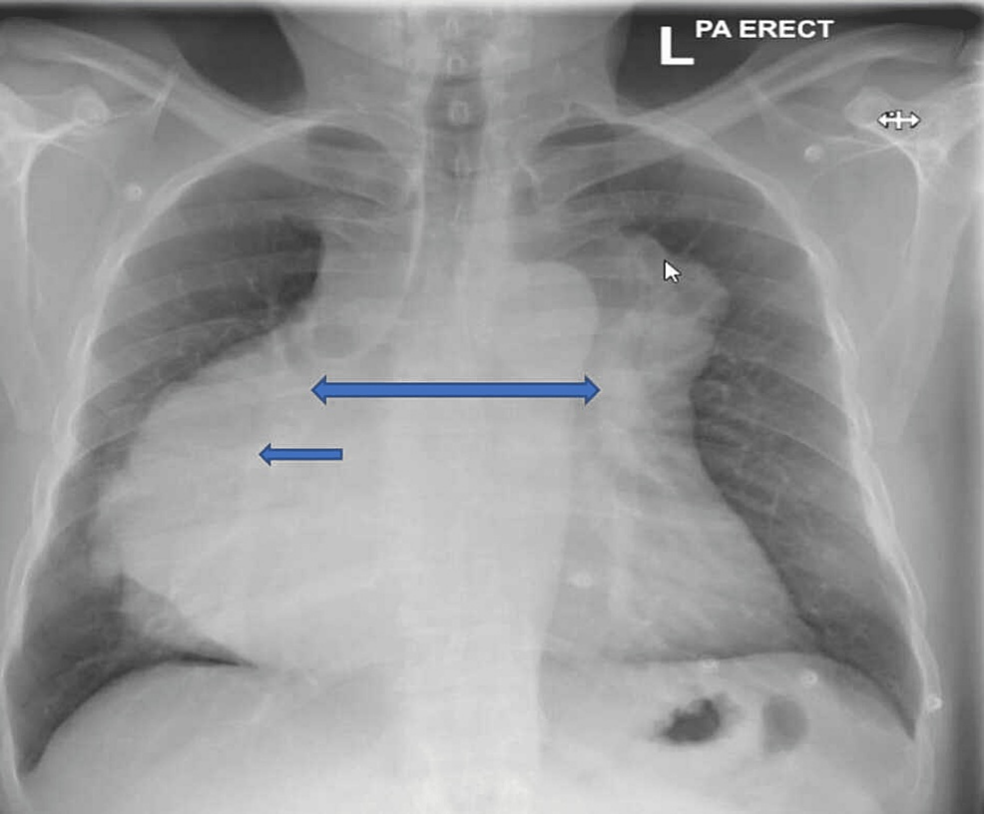
CXR showing a right-sided mediastinal mass and widening of the mediastinum (blue arrows) CXR: chest radiograph

The chest radiograph (CXR) showed a right-sided mediastinal mass (Figure 1). A repeat echocardiogram two days later showed a moderate to large pericardial effusion showing some features of tamponade, which was drained (Videos 4-8).

**Video 4:** Echocardiogram (apical four-chamber view) showing a large pericardial effusion around the left ventricle

**Video 5:** Echocardiogram (apical four-chamber view) showing a large pericardial effusion around the right ventricle, atrium, and left ventricle

**Video 6:** Echocardiogram (PLAX view) showing a large pericardial effusion PLAX: parasternal long-axis view

**Video 7:** Repeat echocardiogram (PLAX view) showing reduction in pericardial effusion size post pericardiocentesis PLAX: parasternal long-axis view

**Video 8:** Repeat echocardiogram (apical four-chamber view) showing reduction in pericardial effusion size post pericardiocentesis

**Figure 2:**
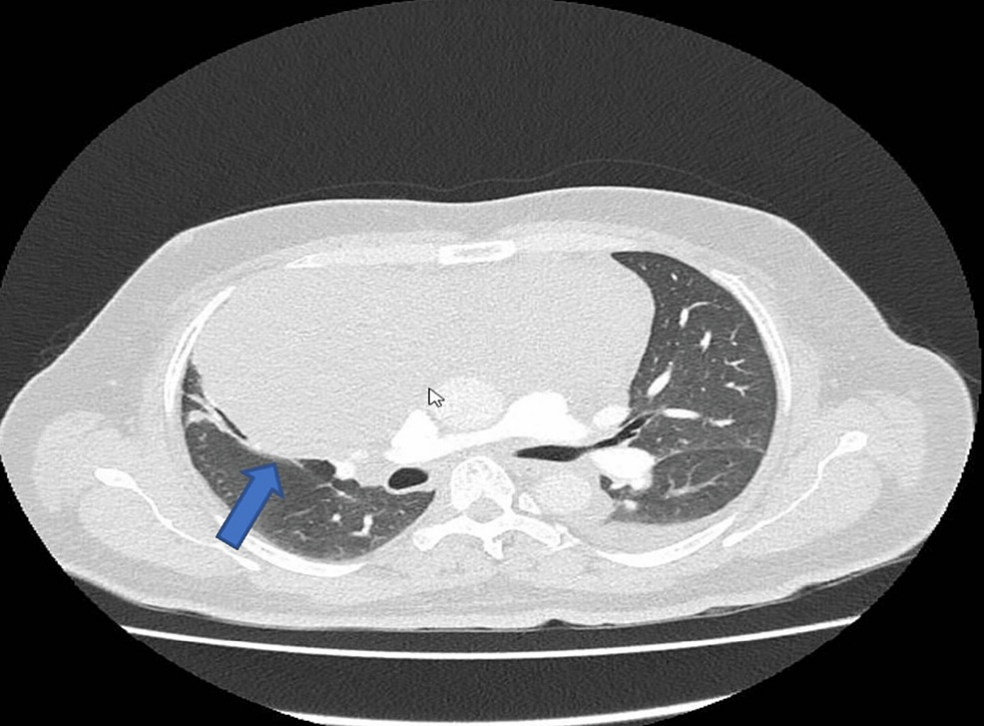
Computed tomography pulmonary angiogram showing a right-sided mediastinal mass (blue arrow) and a small left-sided pleural effusion

The pericardial effusion cytology showed moderately cellular fluid preparations containing blood, small lymphocytes, and macrophages, but no malignant cells were seen. Computed tomography pulmonary angiography (CTPA) showed a large right-sided mediastinal mass without any evidence of pulmonary embolism (Figure 2).

A computed tomography scan of the chest, abdomen, and pelvis showed simple renal cysts and left brachiocephalic and superior vena cava thrombus, and he was commenced on treatment dose tinzaparin. A positron emission tomography (PET) scan showed a moderately avid anterior mediastinal mass, with uptake less than the liver background and no evidence of metastasis. The patient was ineligible for peptide receptor radionuclide therapy (PRRT). Ultrasound scan of the neck showed multiple small lymph nodes with the largest measuring no more than 7 mm in size in any dimension, and biopsies were obtained from it, which were consistent with a carcinoid tumor. The patient was reviewed by hematology and oncology teams and was commenced on prednisolone 5 mg once daily (OD) and omeprazole 20 mg OD. In addition, he was also commenced on cholecalciferol 1,000 units OD for vitamin D deficiency, metoprolol 25 mg twice daily for SVT/chest pain, tinzaparin 14,000 units OD for SVC thrombus, and allopurinol 300 mg OD prior to initiating chemotherapy.

Following the multidisciplinary team (MDT) discussion, he was commenced on neoadjuvant treatment fluorouracil, carboplatin, and streptozocin (FCarboStrep) as the tumor was found to be locally advanced, currently unresectable mediastinal atypical carcinoid, and Ki67 was 5%-6%. The patient developed chest pain, fatigue, and shortness of breath after the first chemotherapy cycle. He became quite unwell with similar symptoms a day after the second cycle and was diagnosed with lower respiratory tract infection and was prescribed Co-amoxiclav 625 mg thrice daily for seven days. He was then commenced on a 5-fluorouracil regimen including carboplatin and streptozocin. He still experienced chest pain radiating to his jaw on chemotherapy, which was likely due to the spasmodic effect of 5-fluorouracil chemotherapy. A repeat echocardiogram did not show normal left ventricular ejection fraction with no regional wall motion abnormalities (RWMA). He is on ongoing chemotherapy with an aim to offer future surgical intervention, and he is currently tolerating the treatment well.

## Discussion

The majority of typical carcinoid tumors occur in the central airways, leading to airway obstruction due to recurrent pneumonia, and account for about 2% of the total lung tumors [7]. Carcinoid tumors are most commonly found in the GIT but can also be seen in other organs such as the lungs, larynx, bronchus, liver, pancreas, kidneys, ovaries, prostate, and thymus [5]. The reported incidence of carcinoid tumors in the USA is 0.15%, in England is 0.79%, and in Scotland is 1.46% [5,8]. NETs are epithelial neoplasms, and mediastinal NETs account for no more than 5% of the total NETs and have an estimated incidence of one per five million people [9,10]. Mediastinal tumors are mostly located in the anterior mediastinum, although cases in which tumors were located in the middle and posterior mediastinum have also been previously reported [9,10]. Thymus tumors can be typical or atypical, and typical carcinoid tumors histologically have uniform epithelial cells with basophilic cytoplasm, salt and pepper-like chromatin features, and diffuse expression of neuroendocrine markers on immunohistochemistry [10]. The mitotic rate of typical carcinoid is less compared to atypical carcinoid tumors of the mediastinum, and most tumors are not encapsulated and can grow aggressively. Almost half of the typical carcinoid patients have localized symptoms such as pain, cough, and shortness of breath, and about 30% of patients have features of paraneoplastic syndrome due to additional hormonal production, resulting in Cushing’s syndrome with and without cutaneous hyperpigmentation, and also produce parathyroid hormone-like substances, resulting in hypercalcemia and hyperphosphatemia. Occasionally, it may also result in primary hyperparathyroidism in the context of multiple endocrine neoplasia type 1 (MEN-1) syndrome [10]. About 50% of patients with typical carcinoid tumors show local or distal metastasis, and bones and the lungs are the two most frequently involved sites. Surgical resection is the therapy of choice in operable typical carcinoid tumors, and there is a lack of reliable data on the role of chemotherapy and radiotherapy [10,11].

Atypical carcinoids on the other hand account for about 40%-50% of the total thymus mediastinal NETs, and they differ from the typical carcinoid tumors due to their slightly increased mitotic rate and focal necrosis [10]. Atypical carcinoid tumors mostly affect middle-aged adults aged 48-55 years and account for about 40%-50% of all thymus NETs [10,12,13]. The genetic alterations of typical carcinoid tumors overlap with atypical carcinoid, and at least 25% of atypical carcinoid tumors have metastasized to mediastinal, cervical, or supraclavicular lymph nodes at the time of diagnosis. The five-year survival rate of atypical carcinoid tumors is slightly worse compared to typical carcinoid tumors [10]. Several patient case reports of atypical carcinoid tumors have been published in the past, and the presentation varies depending on the site involved [6,14,15].

Carcinoid tumors are quite picked up as incidental findings when patients present to the hospital for other reasons. Xuan et al. [6] reported a case report on a patient who presented with a one-month history of worsening chest pain on exertion followed by cough and hemoptysis after 11 days, and high-resolution computed tomography (HRCT) showed a soft tissue mass in the left anterior mediastinum. The patient was treated with chemotherapy and radiotherapy, and surgery was rejected by the patient’s family [6,14]. Kosmas et al. [14] reported a case of a 66-year-old male patient who presented to the outpatient clinic with dyspnea and fatigue for a fortnight, and HRCT showed a mediastinal tumor located in the anterior upper mediastinum. Fluorodeoxyglucose-positron emission tomography (FDG-PET) scan did not show any metastasis of the disease, and cytology confirmed the presence of a neuroendocrine tumor that was surgically excised. The patient was diagnosed with an intermediate-grade atypical carcinoid tumor based on the WHO classification and did not receive any chemotherapy as part of the treatment.

Early diagnosis is key to the management of NETs, and neoadjuvant chemotherapy should be considered in certain cases, although most tumors are unresectable at the time of diagnosis. Nevertheless, the overall prognosis and five-year survival for patients diagnosed with NETs have improved, and follow-up should include CT scans and hormonal assessment. Patients with carcinoid tumors should have 6-12 monthly regular follow-ups to monitor for carcinoid-related heart disease and adjust therapy if required [1].

## Conclusions

In conclusion, carcinoid tumors are rare but aggressive neuroendocrine tumors that originate mainly in the gastrointestinal tract but can also originate in the lungs, ovary, and mediastinum. Patients with atypical carcinoids may be asymptomatic or may present with a wide range of symptoms. Patients presenting with supraventricular tachycardia secondary and clinically significant pericardial effusion have never been reported before, and this is the first case, to the best of our knowledge. It is therefore important that patients presenting with pericardial effusion get a further assessment to rule out serious underlying conditions.
